# Upper limb impairments associated with spasticity in neurological disorders

**DOI:** 10.1186/1743-0003-4-45

**Published:** 2007-11-29

**Authors:** Cheng-Chi Tsao, Mehdi M Mirbagheri

**Affiliations:** 1 Department of Physical Medicine and Rehabilitation, Northwestern University, Chicago, USA; 2 Sensory Motor Performance Program, Rehabilitation Institute of Chicago, Chicago, USA

## Abstract

**Background:**

While upper-extremity movement in individuals with neurological disorders such as stroke and spinal cord injury (SCI) has been studied for many years, the effects of spasticity on arm movement have been poorly quantified. The present study is designed to characterize the nature of impaired arm movements associated with spasticity in these two clinical populations. By comparing impaired voluntary movements between these two groups, we will gain a greater understanding of the effects of the type of spasticity on these movements and, potentially a better understanding of the underlying impairment mechanisms.

**Methods:**

We characterized the kinematics and kinetics of rapid arm movement in SCI and neurologically intact subjects and in both the paretic and non-paretic limbs in stroke subjects. The kinematics of rapid elbow extension over the entire range of motion were quantified by measuring movement trajectory and its derivatives; i.e. movement velocity and acceleration. The kinetics were quantified by measuring maximum isometric voluntary contractions of elbow flexors and extensors. The movement smoothness was estimated using two different computational techniques.

**Results:**

Most kinematic and kinetic and movement smoothness parameters changed significantly in paretic as compared to normal arms in stroke subjects (p < 0.003). Surprisingly, there were no significant differences in these parameters between SCI and stroke subjects, except for the movement smoothness (p ≤ 0.02). Extension was significantly less smooth in the paretic compared to the non-paretic arm in the stroke group (p < 0.003), whereas it was within the normal range in the SCI group. There was also no significant difference in these parameters between the non-paretic arm in stroke subjects and the normal arm in healthy subjects.

**Conclusion:**

The findings suggest that although the cause and location of injury are different in spastic stroke and SCI subjects, the impairments in arm voluntary movement were similar in the two spastic groups. Our results also suggest that the non-paretic arm in stroke subjects was not distinguishable from the normal, and might therefore be used as an appropriate control for studying movement of the paretic arm.

## Introduction

The movement impairments following neurological illness such as stroke and spinal cord injury are caused by disturbances in descending commands, although the precise mechanisms by which disrupted commands affect voluntary function are uncertain. However, several mechanisms including abnormal muscle recruitment, weakness and spasticity have been suggested as contributing factors [1,2]. Spasticity is a motor disorder associated with lesions at different levels of the nervous system. It can directly or indirectly change mechanical properties of the neuromuscular system, particularly in chronic patients, and has been linked to impaired voluntary movement through different mechanisms [3-7].

It is possible that the nature of the movement impairments are different in spastic subjects with different etiologies of spasticity, such as between stroke and SCI. For example, a combination of upper motor neuron and lower motor neuron impairment may occur in many cervical SCI patients where the anterior horn cells at the site of injury are injured and may dampen the magnitude of the normal spastic response at this level, thereby diminishing spastic resistance to the movement. Therefore, comparison of impaired voluntary movement between stroke and SCI groups is warranted to understand possible effects of the etiology of spasticity on the nature of these impairments and their underlying mechanisms.

Previous studies have focused on reaching and grasping movements for individuals with stroke or SCI [8-10]. The effects of spasticity on elbow movement, however, have not been fully characterized. In stroke, although some kinematic parameters of the spastic arm have been measured [11-13], some unresolved issues remain. First, elbow movement has been described over only a narrow portion of its range of motion (ROM). To fully characterize impairments it is critically important to examine elbow joint movement over the entire ROM since mechanical abnormalities of spastic joints are maximized at the extremes of the ROM, as shown previously [5,14]. Secondly, although lack of or reduced smoothness is a major problem in voluntary arm movement, previous studies have focused on distal (i.e., hand) movement [15], with little information available on the smoothness of movement trajectories at the elbow.

Experimental studies on arm movement dysfunction in patients with SCI have focused on functional limitations caused by contracture or paralysis of the arm [16,17]. Compared to stroke, even less quantitative information exists regarding the performance of the spastic elbow in patients with SCI is available, perhaps due to difficulty accessing appropriate subjects.

In the present study, we were interested in addressing these deficits and testing whether impairments in voluntary arm movement differed in patients with different origins of spasticity (in this study, subjects with stroke and SCI). By comparing the impaired voluntary movement between these two groups, we sought to gain a greater understanding of the effects of the type of spasticity (i.e. spinal or cerebral) on these movements. To fully characterize different kinematic, kinetic and movement smoothness parameters, we quantified voluntary full flexion/extension movements of the elbow at maximum speed. Full range of motion and maximum speed are required to elicit certain movement impairments, such as reduced smoothness. We postulated the existence of several different abnormalities in upper extremity kinematics in subjects with stroke versus SCI, in paretic versus non-paretic arms of hemiparetic stroke survivors, and in SCI versus healthy subjects.

## Methods

### Subjects

Patients with paretic arms, ten due to stroke, and eight due to incomplete SCI; and 10 healthy subjects were recruited to participate in this study. The inclusion criteria for stroke subjects were stable medical condition, absence of expressive or receptive aphasia, absence of sensory or motor neglect in the paretic arm, absence of muscle tone abnormalities in the non-paretic arm, absence of motor or sensory deficits in the non-paretic arm, absence of severe muscle wasting or sensory deficits in the paretic arm, spasticity present in the paretic arm, and at least 12 months post-stroke. The inclusion criteria for SCI subjects were traumatic, non-progressive SCI with an American Spinal Injury Association (ASIA) impairment scale classification of C or D indicating motor incomplete lesions, neurological level of C4–C5, spasticity present in the arm, and minimum 1 year post-injury.

Healthy subjects with a mean age of 45 ± 12.3 SD years were age-matched to the stroke and SCI subjects (49.7 ± 10.2 SD years and 42 ± 8.3 SD, respectively), and with no history of neuromuscular disease served as controls. All the subjects gave informed consent to the experimental procedures, which had been reviewed and approved by the Institutional Review Board of Northwestern University.

### Clinical assessment

Stroke survivors and SCI subjects were assessed clinically prior to each experiment using the modified 6-point Ashworth scale (MAS) to assess muscle tone (see Table 1) [18,19].

**Table 1:** Modified Ashworth Scale – (MAS) [18]

**Grade**	**Description**
0	No increase in muscle tone
1	Slight increase in muscle tone, manifested by a catch and release or by minimal resistance at the end of the range of motion (ROM) when the affected part(s) is moved in flexion or extension.
1+	Slight increase in muscle tone, manifested by a catch, followed by minimal resistance throughout the remainder (less than half) of the ROM.
2	More marked increase in muscle tone through most of the ROM, but affected part(s) easily moved.
3	Considerable increase in muscle tone, passive movement difficult.
4	Affected part(s) rigid in flexion or extension.

In SCI subjects with incomplete motor function loss, the sides of the body are often affected differently, so, both sides were assessed in this study. The side with the highest modified Ashworth scale and lowest isometric maximum elbow extension torque, which were always on the same side, was studied.

The MAS scores varied between 1 and 4 in both stroke and SCI groups.

### Apparatus

**Figure 1:**
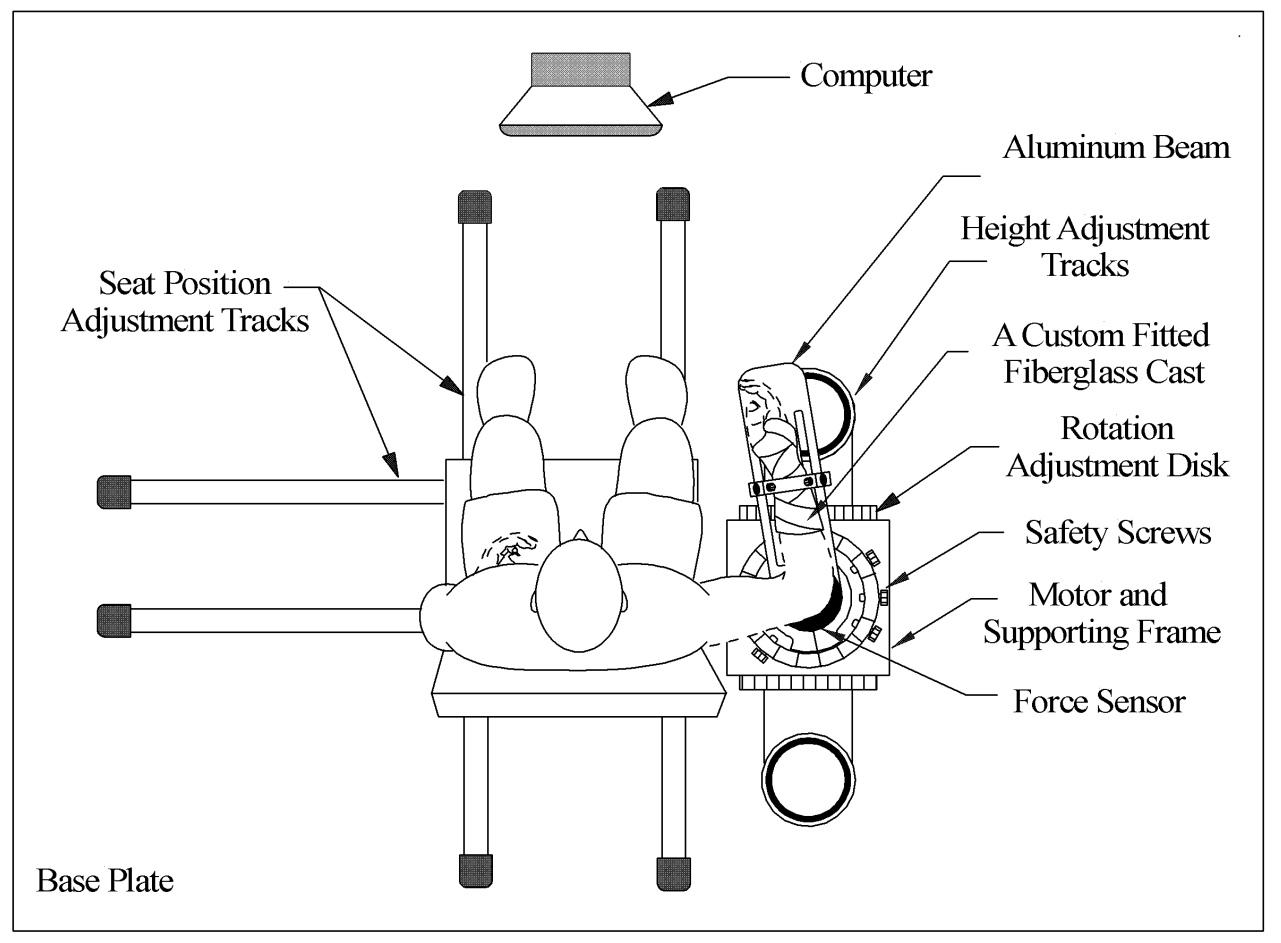
The apparatus including the height adjustable chair, and force and position sensors.

Figure 1 shows a schematic diagram of the apparatus. Subjects were seated and strapped to an adjustable experimental chair with the forearm attached to the beam of the apparatus mounted on a torque cell by a custom fitted fiberglass cast. The seat was adjusted to provide a shoulder abduction of 80 degrees. The axis of elbow rotation was aligned with the axis of the torque sensor and potentiometer.

### Procedures

Subjects were asked to move their forearm from full elbow flexion to extension at maximum speed, with shoulder flexion angle was set at zero degrees. These movements were recorded 5 times and ensemble-averaged. We found that 5 trials provided a strong estimate of mean movement performance, since the typical standard deviation of the movement trajectory was less than 10%.

The elbow position and the torque were measured with a precision potentiometer and torque transducer. Displacements in the flexion direction were taken as negative and those in the extension direction as positive. An elbow angle of 90 degrees was considered the Neutral Position (NP) and defined as zero. Torque was assigned a polarity consistent with the direction of the movement that it would generate (i.e. extension torque was taken as positive).

Both the paretic and non-paretic arms were assessed in individuals with stroke, more affected arm in individuals with SCI (spastic SCI) and the dominant arm in the healthy controls (normal).

### Data analysis

#### Kinematics

**Figure 2:**
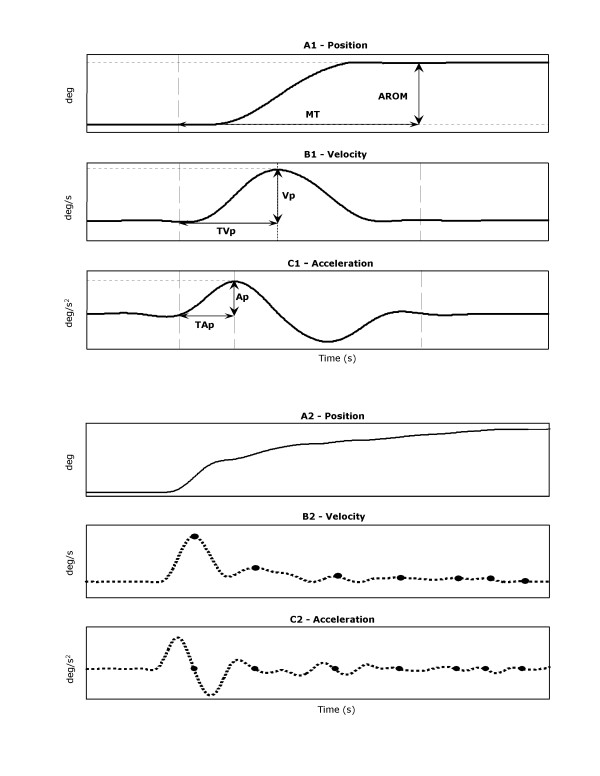
A typical movement trajectory of rapid elbow extension generated by a normal and a stroke subject. Normal: **A1 **Position; **B1 **Velocity; and **C1 **Acceleration. Stroke: **A2 **Position; **B2 **Velocity; and **C2 **Acceleration. Circles in **B2, C2 **represent zero-crossings in the acceleration. MT: movement time, AROM: active range of motion, Vp: peak velocity, Ap: peak acceleration, TVp: the latency to peak velocity, TAp: the latency to peak acceleration.

Angular velocity and acceleration were calculated from the first and second derivatives of the elbow angular position data (Figure 2), respectively. The position, velocity, and acceleration data were used to quantify kinematic parameters: i.e., peak angular velocity (Vp), latency to peak angular velocity (TVp), peak angular acceleration (Ap), latency to peak acceleration (TAp), movement time (MT), active range of motion (AROM), and movement smoothness. The MT, AROM, onset and end of an elbow extension were determined from the velocity profile. The onset and end of each movement were defined as the first sample with velocity larger and smaller, respectively, than 5% of Ap [20].

#### Kinetics

All study participants were asked to generate an isometric maximum voluntary contraction (MVC) in the direction of elbow extension at the NP for 5 seconds. The process was performed 3 times and measurements were averaged.

#### Movement smoothess

Impaired voluntary movements of spastic arms are characterized by the loss of smoothness in the movement trajectory [13,21,22]. In healthy subjects movement trajectories are smooth (Fig. 2-A1) with single-peaked, bell-shaped velocity profiles (i.e. single acceleration phase followed by a single deceleration phase) (Fig. 2-B1). In contrast, movement trajectories from spastic subjects are rippled (Fig. 2-A2), and with multiple peaks and irregularities in both velocity (Fig. 2-B2) and acceleration (Fig. 2-C2). Two major computational methods were used to measure movement smoothness.

##### Number of movement unit (NMU)

The NMU of the movement trajectory was defined as the total number of velocity peaks between the onset and offset of the movement [23] (Fig. 2-B2). A velocity peak was identified in the acceleration profile as the point where the trajectory crossed the zero line and the sign of acceleration changed from positive (accelerating) to negative (decelerating) as shown in Fig. 2-C2.

##### Normalized jerk score (NJS)

The NJS was computed from the jerk, which was defined by Kitazawa, *et al.*[24]as the third derivative of the angular position, used as the index of trajectory smoothness. It successfully captures the jerkiness of reaching movements in monkeys with limb ataxia [24]. The NJS was calculated from Equation-1:

NJS=sqrt{1/2∗∫t1t2(d3p/dt3)2∗dt∗[t5/(Pt2−Pt1)2]}
MathType@MTEF@5@5@+=feaafiart1ev1aaatCvAUfKttLearuWrP9MDH5MBPbIqV92AaeXatLxBI9gBaebbnrfifHhDYfgasaacPC6xNi=xI8qiVKYPFjYdHaVhbbf9v8qqaqFr0xc9vqFj0dXdbba91qpepeI8k8fiI+fsY=rqGqVepae9pg0db9vqaiVgFr0xfr=xfr=xc9adbaqaaeGacaGaaiaabeqaaeqabiWaaaGcbaGaemOta4KaemOsaOKaem4uamLaeyypa0Jaem4CamNaemyCaeNaemOCaiNaemiDaqNaei4EaSNaeGymaeJaei4la8IaeGOmaiJaey4fIOYaa8qCaeaacqGGOaakcqWGKbazdaahaaWcbeqaaiabiodaZaaakiabdchaWjabc+caViabdsgaKjabdsha0naaCaaaleqabaGaeG4mamdaaOGaeiykaKYaaWbaaSqabeaacqaIYaGmaaGccqGHxiIkcqWGKbazcqWG0baDcqGHxiIkcqGGBbWwcqWG0baDdaahaaWcbeqaaiabiwda1aaakiabc+caViabcIcaOiabdcfaqnaaBaaaleaacqWG0baDcqaIYaGmaeqaaOGaeyOeI0Iaemiuaa1aaSbaaSqaaiabdsha0jabigdaXaqabaGccqGGPaqkdaahaaWcbeqaaiabikdaYaaakiabc2faDjabc2ha9bWcbaGaemiDaqNaeGymaedabaGaemiDaqNaeGOmaidaniabgUIiYdaaaa@65B7@

where

*P*_*i*_: Elbow angular position at the i^th ^sample

*t*_1_: Onset of movement

*t*_2_: Offset of movement

*d*^3^*p*/*dt*^3^: Third derivative of the angular position data

*t*: Movement time

*P*_*t*2 _- *P*_*t*1_: AROM

### Statistical analysis

Non-parametric Wilcoxon rank tests were used for group comparisons (e.g. paretic versus non-paretic limbs in stroke, paretic limbs in stroke versus spastic limbs in SCI; non-paretic limbs in stroke versus normal limbs; spastic SCI limbs versus normal limbs). We used Wilcoxon matched pairs signed rank sum test for the comparison of the two sides of the stroke subjects, and Wilcoxon signed rank sum test for the comparison between arm measurements in other groups. All statistical analyses were performed using SAS statistical software (SAS 9.1.3. SAS Inc. Cary, NC). A Bonferroni correction was used to adjust the alpha level for all our statistical comparisons. We made four group comparisons, therefore, a significance level was set at 0.013 (= 0.05/4).

Spearmen correlation coefficients were computed to test the relationship between the kinematic, kinetic and movement smoothness measures and Ashworth scores in the paretic and spastic SCI arms.

## Results

### Paretic versus non-paretic arm in stroke subjects

**Figure 3:**
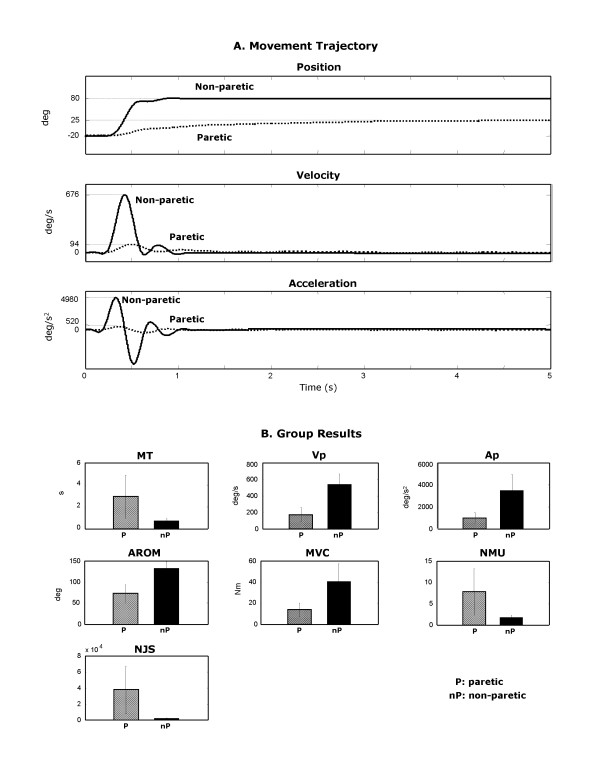
**A **Movement trajectories of elbow angular position, velocity and acceleration of the paretic arm (dotted-line) and the non-paretic arm (solid-line) in a typical stroke subject; **B **Kinematic, kinetic and smoothness parameters which are significantly different between the paretic and non-paretic arms: MT: movement time; Vp: Peak velocity; Ap: peak acceleration; AROM: active range of motion; MVC: isometric muscle strength of elbow extensors; NJS: normalized jerk score; NMU: number of movement unit. Group average ± Standard deviation.

We quantified the impairments during the rapid elbow extension movement of the spastic upper limb. Figure 3A shows the movement trajectory (top panel), velocity (middle panel), and acceleration (bottom panel) for the paretic and the non-paretic arm in a representative stroke survivor. For the paretic arm, the AROM was 60 degrees (60%) smaller, and MT was 3 seconds (approximately 4 times) longer than that of the non-paretic arm. The peak velocity and peak acceleration were approximately 85% and 90% smaller, respectively, in the paretic than in the non-paretic arm.

Figure 3B shows the group means and standard deviations of the kinematic, kinetic and smoothness parameters for paretic and non-paretic arms. Impairments were evident in most of these parameters. MT was significantly longer, Vp and Ap smaller, AROM smaller, and MVC lower (each at *p *< 0.001). There was no significant difference (p > 0.1) between paretic and non-paretic arms in latencies to peak velocity and acceleration (TVp, TAp).

Movement of the paretic arm was jerky, indicated by the ripples on the movement trajectory, velocity, and acceleration graphs (Figure 3A). The group results show that NMU and NJS were significantly larger in the paretic than the non-paretic arm (*p *< 0.01). The NMU and NJS were more than 4 and 8 times larger, respectively, in the paretic than in the non-paretic arm indicating jerky movement.

### Non-paretic arm versus normal arm

**Figure 4:**
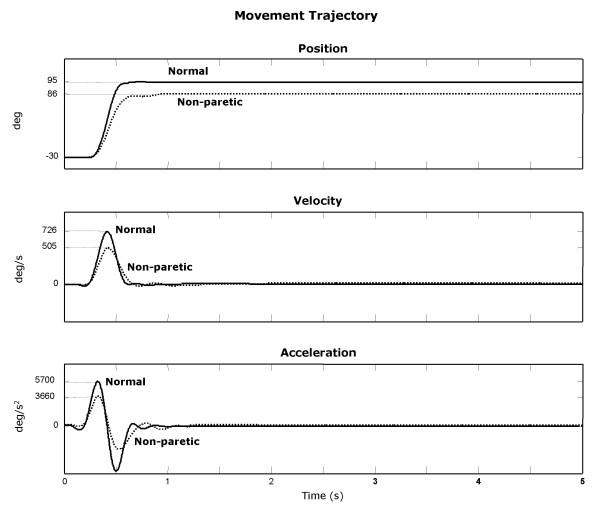
Movement trajectories of elbow angular position, velocity and acceleration of the non-paretic arm (dotted-line) in a typical stroke subject and the normal arm in a typical healthy subject (normal; solid-line).

It has been suggested that the non-paretic limb can be influenced to some extent by stroke [25,26]. We probed this claim in elbow extension movement by comparing the kinematics, kinetics and smoothness parameters of non-paretic elbow movement to those of healthy subjects (Normal). Figure 4 shows typical movement trajectories of non-paretic and normal arms. The non-paretic arm showed a slightly slower movement and less smooth trajectory than the normal arm. In the non-paretic arm, AROM was ~8% smaller, MT was ~45% longer, and Vp and Ap were ~30%, ~36% lower, respectively. The position trajectory for the non-paretic arm and its related velocity and acceleration had a small but typical extra ripple, indicating a mild jerkiness. Although the non-paretic arms seem to show mild impairments in the movement trajectory, there were no significant differences in kinematic and kinetic parameters between the non-paretic and normal groups (p > 0.11). These findings suggest that although the non-paretic arm is not entirely "normal", it may be considered as a suitable control to eliminate the effects of inter-subject variability.

### Spastic arm in SCI versus normal arm

**Figure 5:**
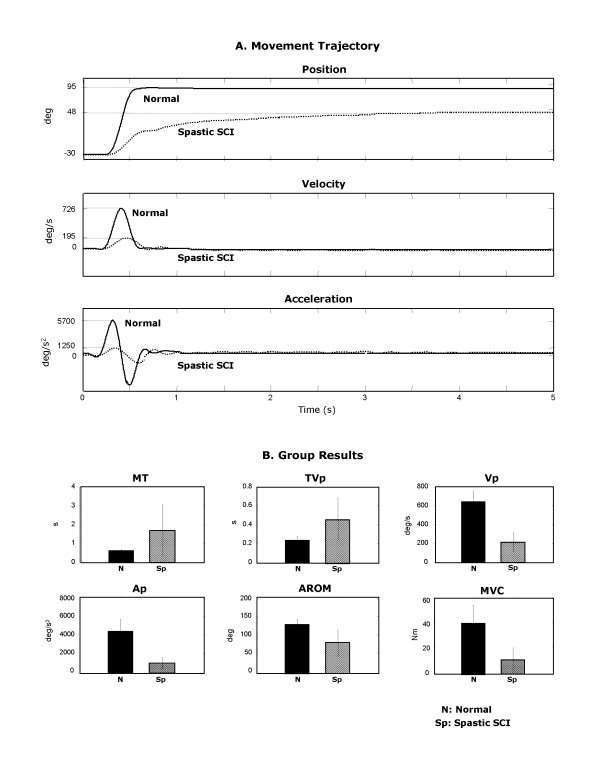
**A **Movement trajectories of elbow angular position, velocity, and acceleration of the spastic arm in a typical SCI subject (spastic SCI; dotted-line) and the normal arm in a typical healthy subject (Normal; solid-line); **B **Kinematic, kinetic and smoothness variables which are significantly different between the spastic SCI and Normal arms: MT: movement time; TVp: latency to peak velocity; Vp: Peak velocity; Ap: peak acceleration; AROM: active range of motion; MVC: isometric muscle strength of elbow extensors. Group average ± Standard deviation.

Representative movement trajectories of spastic arms in subjects with SCI and normal arms are shown in Figure 5A. In SCI subjects, AROM was ~42% smaller, MT was approximately 7 times longer and Vp and Ap were over 70% smaller.

The group results indicate that all these kinematic parameters were significantly changed in the spastic SCI arm (Figure 5B, p < 0.01). Furthermore, MVC in the spastic SCI arm was significantly smaller than in the Normal arm (p < 0.01). There were no significant differences in other movement parameters (p > 0.1).

Mild jerkiness was also evident in the subject with SCI as an extra ripple in the graphs of movement trajectory, velocity and acceleration (Figure 5A). However, there was no significant difference in the smoothness measures between the group results of the spastic SCI and healthy subjects (p > 0.21).

### Paretic arm in stroke versus spastic arm in SCI

**Figure 6:**
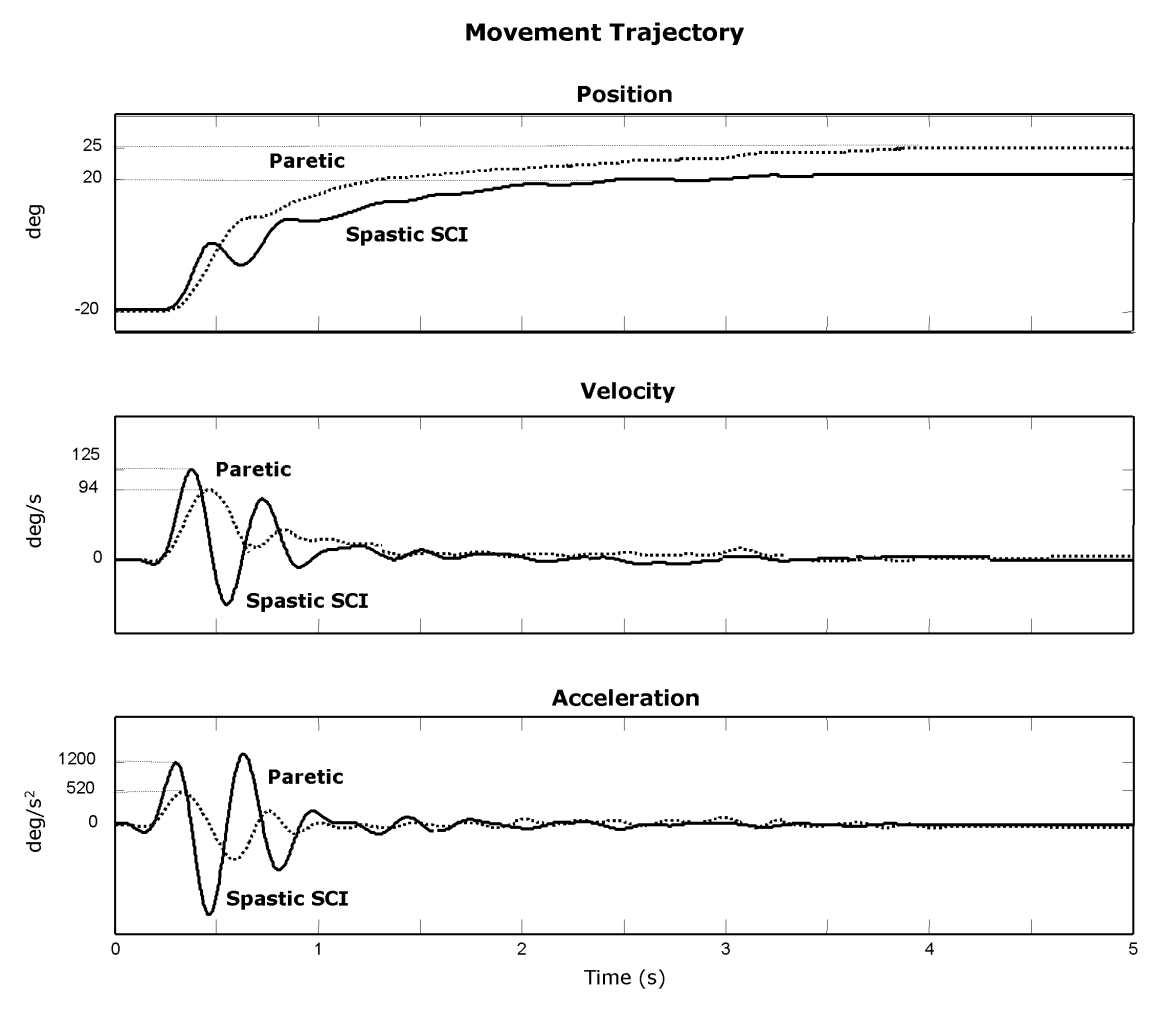
Movement trajectories of paretic in stroke (dotted-line) and of spastic in SCI (solid-line) arms.

Figure 6 shows typical movement trajectories of the paretic arm in stroke and the spastic arm in SCI. Movement impairments, including long MT, small Vp and Ap and jerky movements were evident in both paretic and spastic SCI arms. However, there were no significant differences in kinematics, kinetics, or movement smoothness in the group results for these two patient populations (p > 0.17).

#### Descriptive subgroup analysis

There was no significant difference in movement impairments between the paretic arm and the spastic SCI arm. However, movement trajectories of the paretic arm seemed less smooth than the spastic SCI arm. In an attempt to possibly detect the reduced smoothness, we computed the AROM generated during the first movement unit (AROM_1MU), and the percentage of AROM covered by the first movement unit (%AROM_1MU) in paretic and spastic SCI arms. These two measures indicate a person's ability to precisely scale movement velocity and muscle forces such that a task can be accomplished in a single accelerating and decelerating cycle [23]. If the task can be completed at the first attempt, further minor adjustments of the arm are not needed; the overall movement is continuous and smooth. Therefore, AROM_1MU and %AROM_1MU may also provide an alternative to characterize movement smoothness and help differentiate impairments in voluntary control between paretic and spastic SCI groups.

To eliminate the effect of the large inter-subject variability observed in paretic and spastic SCI groups, patients were assigned to either a "Good Performance" group (G) or a "Fair-Poor Performance" (FP) group by comparing individual values of MT, AROM, MVC, Vp, and AROM_1MU to the group means. If the value of a particular parameter was larger (for AROM, MVC, Vp, and AROM_1MU) or smaller (for MT) than the group mean, that parameter was coded 1, otherwise coded 0. Coding scores from these five parameters were added for each subject to form a sum score. A subject was assigned to the G group if his/her sum score was greater than the group median score (the median of the sum scores of the whole group) and to the FP group if his/her sum score was equal to or smaller than the group median score. Kinematic, kinetic and smoothness parameters were compared between paretic and spastic SCI arms in each performance group.

In the G group, there were no significant differences between the paretic and spastic SCI arms. In the FP group, AROM_1MU and %AROM_1MU were significantly larger for spastic SCI arms than paretic arms (*p ≤ 0.02*), but there were no significant differences in other parameters.

### Correlation between movement and clinical measures

We found no significant correlations (r < 0.5) between the Ashworth scores and our objective measures of voluntary movement.

## Discussion

This study clarifies several important issues regarding impaired voluntary movement of the spastic elbow in patients with neurological disorders. In particular we characterized the nature of the impairments in voluntary movement of two spastic populations.

A number of new insights into movement impairments were provided by this study. First, most kinematic and kinetic parameters were significantly changed in the paretic arm in stroke and the spastic arm in SCI. In addition, the effective methods for measuring movement smoothness were determined; differences in movement smoothness between patients following stroke and SCI were evident *only *when subjects with fair to poor performances (FP) were compared. Interestingly, clinical measures of spasticity (i.e., Ashworth scores) were not related to these objective, voluntary movement parameters. Finally, abnormal kinematics for the non-paretic limb of patients post-stroke indicated a degree of abnormality. However, these changes were not significant, suggesting that the non-paretic limb might be an appropriate control for the paretic arm as it eliminates the effects of inter-subject variability.

Taken together, our findings provide a better understanding of the nature of movement impairments associated with spasticity in patients following stroke and SCI. The similarities and differences in the kinematics and kinetics of the non-paretic and healthy arm provide necessary information for the design and execution of movement studies in stroke subjects.

### Impaired voluntary movement in the paretic arm: kinematics and kinetics

We provide details of movement impairments throughout the *full angular range *of motion of the elbow, recorded at maximum movement speed, a movement and speed range not examined in earlier studies. Differences in the experimental apparatus used for studying single- and multi-joint movements introduce inconsistencies between our work and other studies [15,22,27]. However, the main features of our comparisons between paretic and non-paretic arms are in agreement with earlier studies.

In addition to supporting other research findings that the paretic arm moves more slowly and is profoundly weaker than the non-paretic arm [15,28,29], we provided further insight into the nature of impairments of voluntary movement in stroke subjects by measuring the most important kinematic and kinetic parameters. We also quantified the movement smoothness of the arm trajectory using two different computational measures. Our findings confirm previous research [15,22,30] that the paretic arm accelerates and decelerates multiple times during a single movement, causing jerky movement in the paretic arm.

#### Mechanisms underlying impaired movement in stroke

Although the precise underlying mechanisms by which disrupted descending cortical input affects voluntary function are uncertain, three major mechanisms have been suggested as contributing factors to movement impairment: abnormal muscle recruitment, weakness, and altered spinal reflexes, specifically spasticity [1,2].

Abnormal muscle recruitment, in which a voluntary movement is performed by synergistic patterns, can either directly or indirectly lead to impairment by limiting independent motion of different joints within the impaired limb.

The immobility imposed by a weakness from a decrease in neural drive command can reduce the voluntary force shown in the MVC of the elbow extensors either directly or indirectly. Such immobility may lead to disuse-related muscular atrophy and/or contracture, which is secondary to the loss of active range of motion. Animal studies have shown that reduced neuronal drive leads to muscular atrophy and subsequent physiological changes of the skeletal muscles [31]. The reciprocal inhibitory effects of spastic elbow flexors on the voluntary activation of elbow extensors may also be a cause of abnormal muscle recruitment. This is supported by our findings that the MVC of elbow extensors was significantly lower in spastic than healthy arms (Figure 3B) and inversely correlated with reflex stiffness gain of the elbow flexors [5].

Spasticity may also be affected by the mechanisms discussed earlier and may be linked to impaired function through other mechanisms. Hypertonia causes hyperactivity of spastic muscles that can lead to hypoactivity of their antagonists through reciprocal inhibition [1,2]. These abnormalities may lead to shortening of the muscles resulting in alteration of the muscle length-tension relationship. These combined changes may ultimately lead to impaired movement and function [28,32,33]. Our findings support the relationship between spasticity and impairments in voluntary movement by indicating that the abnormal reduction in Vp and Ap of elbow voluntary extension in the paretic group are strongly correlated with the abnormal modulation of reflex stiffness gain [5]. Furthermore, this relationship has been supported by findings that reducing hypertonia by therapeutic interventions, such as medication and functional electrical stimulation, increases maximum voluntary force [3,7] and improves function [4,7,6].

### Movement smoothness

This study showed multiple phases of acceleration and deceleration are involved in elbow extension of the paretic arm in post-stroke subjects. Decreased smoothness of the movement trajectory was noted in individuals with stroke in the end-point trajectory during goal-directed reaching [13,15,22]. Our findings provide further information on the degraded movement smoothness in the paretic arm by using two different computational methods to quantify smoothness.

NMU, and NJS have been used to quantify the movement smoothness of reaching in individuals with stroke [13,22,34], prenatal brain injury [23], and Parkinson's disease [35]. However, it remains unclear which measurement best detects changes in smoothness. Our results demonstrate that both NMU and NJS were several times greater for paretic than non-paretic arms and are therefore sensitive enough to detect the decrease in smoothness of a movement trajectory. One possible explanation is that NMU and NJS are calculated from the angular velocity, acceleration, and jerk, all of which are higher-order derivatives of the angular position in which changes in position are magnified by differentiation.

#### Mechanisms underlying the decreased smoothness in the stroke arm

Non-smooth movement in reaching may relate to deficits in global movement trajectory planning and in the inability to coordinate multiple joints [27]. Peripheral factors, such as muscle strength, have not been directly related to jerkiness of the end-point trajectory by other researchers [22,34]. In our study, the movement involved a single joint (elbow) with the upper arm fully stabilized so that subjects did not have to overcome any interactions between joints during movement. Subjects were instructed to push out their forearm as fast as possible and to stop when they reached the end of their range of movement. They needed to plan the trajectory to the end point with respect to the spatial location of the target (i.e. the end of the range of motion) to achieve this task. Thus, the non-smooth movement of the paretic arm may be a consequence of impaired trajectory planning in stroke survivors.

Decreased smoothness of elbow extension may be associated with spastic hypertonia of the elbow flexors. Fellows *et al.*[11] found that in stroke subjects, the activation duration and the amplitude of biceps contractions were significantly increased with the peak velocity of elbow extension, which was not present in normal control subjects, suggesting abnormal co-activation pattern of elbow flexors during rapid elbow extension may lead to a non-smooth trajectory. In the current study, we found a correlation between Vp and NMU (r = -0.65, using Spearman correlation coefficients) indicating that the trajectory is more segmented with the slower Vp of stroke survivors. Others have also found Vp and Ap of elbow extension are inversely correlated with the reflex gain of the elbow flexors, indicating the impact of reflex hypertonia on the Vp [5]. Given the correlation between Vp and movement jerkiness reported in this study, the reflex hypertonia of elbow flexors may also contribute to the lower smoothness of the movement trajectory in elbow extension of stroke subjects.

### Can the non-paretic arm be used as control?

Several studies have shown that muscle strength and controllability of the non-paretic arm is reduced in stroke survivors and it therefore, cannot serve as control for evaluating the functional recovery of the paretic arm [25,26]. However, the non-paretic arm offers advantages over a healthy arm as a control primarily because within-subject variability in biomechanical properties is small. Our comparison of movement kinematics in non-paretic and normal arms showed no significant differences between non-paretic and normal arms. However, the statistical power for this comparison was smaller than 40% for the measured parameters indicating a reduced chance of detecting significant differences. Therefore, we can *only *suggest that the non-paretic arm may be considered as control for evaluating the performance of arm movements as it eliminates inter-subject variability effects.

### Movement impairments in different spastic populations

In all our SCI subjects, control of elbow muscles was compromised by an incomplete C4–C5 injury. Compared to normal subjects, the spastic arm of SCI subjects moved more slowly, had a smaller AROM, and a weaker MVC. However, none of the smoothness measures was significantly different from those of the normal arm. These findings are consistent with previous findings that subjects with C4–C6 incomplete SCI can generate smooth elbow movement trajectories, although peak velocity is significantly reduced [36].

The finding of relatively smooth movements in our SCI subjects may relate to the integrity of the cortical motor centers in this population which provide the needed capability in trajectory planning. In contrast to stroke, (where lesions to the brain may reduce the control of movement smoothness), in SCI subjects these parts of CNS are largely intact, resulting in more smooth movement. In addition, however, SCI subjects with C5–C6 injury usually generate reaching movements by activating the agonist muscles alone [37]. In many cervical SCI subjects, the anterior horn cells at the lesion location are injured that may dampen the normal spasticity at this level. Decreased spasticity, and therefore reduced co-activation from the antagonists during elbow extension, may also contribute to a smooth trajectory in our SCI subjects. The contribution of either of these mechanisms for the preserved smoothness trajectories in SCI is unclear.

Unlike the spastic SCI group, smoothness measures (NMU and NJS) in the paretic group were significantly greater in the paretic arm than the non-paretic arm. We therefore expected smoothness measures of the spastic arm in SCI to be significantly different from those of the paretic arm in stroke. However, there was no significant difference in smoothness measures between the two patient groups. Further analysis of the data grouped by level of performance (G, FP) reduced inter-subject variability and showed that in the FP group only, paretic arms had smaller AROM_1MU and %AROM_1MU than spastic SCI arms in subjects of the same performance level, indicating a higher capability in SCI subjects to assemble and scale muscle forces to generate a skillful movement. However, the reduced trajectory smoothness of the paretic arms in the FP group, suggested by their smaller AROM_1MU and %AROM_1MU, was not confirmed by other smoothness measures (NMU and NJS). These showed no significant differences between the paretic arms and the spastic SCI arms in the FP group. When individual data in the FP group were carefully examined, we found two smoothness measures (NMU and NJS) for spastic SCI arms had lower mean values and lower rank scores than those in paretic arms. However, differences were not statistically significant due to the small sample size in the subgroups. Future studies with larger samples will be needed to further explore the differences in trajectory planning and control between the stroke and SCI subjects.

### Correlations between kinematic and kinetic measures and ashworth score

The correlations between our objective measures of impaired voluntary movements and Ashworth scores were poor, although we did accept the global clinical assessment that the subjects were spastic, since this was determined by independent clinical examination. These findings indicate that MAS assessments are relatively unreliable as compared with more objective measures of spasticity. There are at least three major reasons that may explain why Ashworth scores were not well correlated to our objective voluntary measures.

First, the Ashworth scale is a clinical measure designed to assess muscle tone by manipulating the joint and measuring its resistance to imposed movement. This movement begins with the muscles in a quiescent state. It follows that the Ashworth scale may not suitable for measuring active movement; whereas we studied active arm movements.

Second, the Ashworth scale measures overall stiffness of the joint, and this single number cannot provide information regarding the characteristics of movement such as range of motion, movement speed and acceleration, and movement smoothness. It also cannot give us a measure of muscle strength.

Finally, the Ashworth scale is neither objective nor quantitative. It is an ordinal number that represents the existence of tone or at the most the approximate severity of tone, but it can neither characterize the contributions of muscular and/or reflex components to tone nor their modulation with position and velocity of the joint stretch. This limitation is described in earlier publications in spastic subjects with SCI [38] and stroke [39-44].

## Conclusion

Our findings show significant differences in major kinematic, kinetic and movement smoothness parameters between the paretic and non-paretic arm in individuals with stroke. Surprisingly, although the cause and location of injury are different in spastic stroke and SCI subjects, there were no significant differences in the impairments between the two groups, except for the movement smoothness. This suggests that the nature of the impairment in arm movement is similar in these two populations. Smoothness was significantly lower in the paretic compared to the non-paretic arm in the stroke subjects, whereas no significant changes in smoothness were evident in the SCI subjects. However, future studies with larger sample sizes will be needed to further explore this issue. Finally, there was no significant difference between the non-paretic and normal arms suggesting that the non-paretic arm may be used as a suitable control for stroke subjects as it minimizes the adverse effects of inter-subject variability.

## Abbreviations

SCI – spinal cord injury

ROM – range of motion

ASIA – American Spinal Injury Association

NP – neutral position

MVC – maximum voluntary contraction

Vp – peak angular velocity

Ap – peak angular acceleration

TVp – latency to peak velocity

TAp – latency to peak acceleration

MT – movement time

AROM – active range of motion

NMU – number of movement unit

NJS – normalized jerk score

AROM_1MU – AROM generated during the first movement unit

%AROM_1MU – the percentage of AROM covered by the first movement unit

G – Good performance

FP – Fair-Poor performance

## Competing interests

The author(s) declare that they have no competing interests.

## Authors' contributions

CT participated in performing the experiment, analyzing and interpreting the data, and writing the paper. MMM designed the study, supervised data collection and analysis, and participated in interpreting and writing the manuscript. Both authors read and approved the final manuscript.
